# Etiologic Aspect of Sarcoidosis as an Allergic Endogenous Infection Caused by *Propionibacterium acnes*

**DOI:** 10.1155/2013/935289

**Published:** 2013-06-16

**Authors:** Yoshinobu Eishi

**Affiliations:** Department of Human Pathology, Tokyo Medical and Dental University Graduate School, 1-5-45, Yushima, Bunkyo-ku, Tokyo 113-8510, Japan

## Abstract

Sarcoidosis is a systemic granulomatous disease of unknown etiology. *Propionibacterium acnes* is the only microorganism that has been isolated from sarcoid lesions. Many *P. acnes* have been detected in sarcoid lymph nodes using quantitative PCR and in sarcoid granulomas by *in situ* hybridization. *P. acnes* trigger factor protein causes a cellular immune response only in sarcoid patients and induces pulmonary granulomas in mice sensitized with the protein and adjuvant, but only those with latent *P. acnes* infection in their lungs. Eradication of *P. acnes* by antibiotics prevents the development of granulomas in this experimental model. Although *P. acnes* is the most common commensal bacterium in the lungs and lymph nodes, *P. acnes*-specific antibody detected the bacterium within sarcoid granulomas of these organs. *P. acnes* can cause latent infection in the lung and lymph node and persist in a cell-wall-deficient form. The dormant form is activated endogenously under certain conditions and proliferates at the site of latent infection. In patients with *P. acnes* hypersensitivity, granulomatous inflammation is triggered by intracellular proliferation of the bacterium. Proliferating bacteria may escape granulomatous isolation, spreading to other organs. Latent *P. acnes* infection in systemic organs can be reactivated by another triggering event, leading to systemic sarcoidosis.

## 1. Introduction

Sarcoidosis is one of the best-known systemic granulomatous diseases. Despite intensive investigation, however, the etiology of sarcoidosis has remained unresolved for more than 100 years [1]. Sarcoidosis seems to result from the exposure of a genetically susceptible subject to an environmental agent, and microbial etiologies of sarcoidosis have long been considered based on the clinical similarities to infectious granulomatous diseases [2]. Several epidemiologic mechanisms may underlie the association of an infective agent or agents with the etiology of sarcoidosis, including spatial, seasonal, and occupational clustering [3]. The results of the a case control etiologic study of sarcoidosis (ACCESS) study support an association between selected microbially-rich environments and sarcoidosis [4].

Mycobacterial and propionibacterial organisms are the most commonly implicated etiologic agents based on studies using polymerase chain reaction (PCR) of microbial DNA from these organisms in tissues from sarcoid patients around the world [5–7]. Different studies have produced considerably varying results, however, with microbial DNA detected in 0% to 80% of sarcoidosis tissues and in 0% to more than 30% of control tissues [8, 9]. The failure to detect microbial DNA from these organisms in samples from some sarcoid patients suggests other causes of sarcoidosis in those patients, whereas detection of the microbial DNA in some control samples suggests latent infection of the bacterium.

Immune responses against microbial antigens from these organisms, such as ESAT-6 and KatG peptides from *Mycobacterium tuberculosis* and a recombinant trigger factor protein from *Propionibacterium acnes*, have been examined in sarcoid patients and control subjects [10–12]. Immune responses are frequently detected in sarcoid patients as well as in some nonsarcoid patients and healthy subjects. Latent infection by these organisms complicates the interpretation of the results of these immunologic studies. Unless microbial antigens that cause a specific immune response found only in sarcoid patients can be used to stimulate an immune response, immunologic approaches will not be sufficient to unequivocally confirm that these organisms are causative.

Granuloma formation results from the persistence of a nondegradable product or a hypersensitivity response [13]. The two mechanisms overlap in most infectious diseases because microorganisms act as both foreign bodies and antigens to induce immunologic responses. Granulomas serve as protective mechanism to sequester and degrade the invading agent. The pathologic hallmark of sarcoidosis is an epithelioid cell granuloma; thus, some etiologic agent of sarcoidosis must be present or have been present within the sarcoid granuloma. Histopathological studies are therefore essential to demonstrate mycobacterial or propionibacterial organisms or antigens within sarcoid granulomas to demonstrate an etiologic link between sarcoidosis and these organisms. 

*P. acnes* is so far the only microorganism isolated from sarcoid lesions by bacterial culture [14, 15]. *P. acnes* is an anaerobic, nonspore-forming, gram-positive rod bacterium indigenous to the skin and mucosal surfaces. A series of Japanese studies has provided accumulating evidence for a role of *P. acnes* in sarcoidosis. In this paper, we propose mechanisms of granuloma formation in response to this indigenous bacterium in subjects with sarcoidosis based on our results obtained using histopathological and experimental approaches and introduce a new concept of endogenous infection caused by hypersensitivity to indigenous bacteria. 

## 2. Bacterial Culture

The lung and its draining lymph nodes are the organs most commonly affected by sarcoidosis. As the lung constantly encounters airborne substances, including pathogens, many researchers have considered infection to trigger sarcoidosis and have thus tried to identify possible causative transmissible agents and their contribution to the mechanism of sarcoid granuloma formation [16, 17]. 

In the late 1970s, a large Japanese research project conducted by many clinicians and microbiologists with support by a grant from the Japanese Ministry of Health was organized to seek the pathogens responsible for sarcoidosis. Extensive trials were performed to isolate microorganisms, including bacteria, viruses, and fungi, from tissue samples (especially biopsied lymph nodes) affected by sarcoidosis. Only *P. acnes*, and no other microorganism, was isolated from the large number of samples [14]. *P. acnes* was isolated in culture from biopsy samples of 31 (78%) of 40 lymph nodes from 40 patients with sarcoidosis [15], whereas this indigenous bacterium was also cultured from 20% of 141 control lymph nodes from patients with diseases other than sarcoidosis. The study was repeated twice to confirm that the initial samples had not been contaminated by cutaneous *P. acnes* during biopsy, and the results of both studies were the same.

Ishige et al. cultured peripheral lung tissue and various lymph nodes obtained from patients with diseases other than sarcoidosis [18]. *P. acnes* was isolated from 24 of 43 lungs and 8 of 11 mediastinal lymph nodes, mostly in pure culture. *P. acnes* was isolated from 10 of 20 gastric and 3 of 12 intestinal lymph nodes; intestinal bacteria were also numerous. The number of *P. acnes* cells isolated was usually no more than 500 colony forming units (CFUs)/g in the lungs and lymph nodes. Of 43 lungs from patients without sarcoidosis, only 4 (9%) had exceptionally high numbers of *P. acnes* cells. Random amplified polymorphic DNA analysis was used to compare the DNA of 45 isolates of *P. acnes* from these patients, 39 isolates from sarcoid lymph nodes, and 67 isolates from normal skin, conjunctiva, and intestine. The *P. acnes* strains in the lung and mediastinal lymph nodes differed genetically from those in the skin. Therefore, contamination from the skin during operative or culture procedures seems unlikely. These findings suggest that *P. acnes* normally resides in peripheral lung tissue and mediastinal lymph nodes.

**Figure 1:**
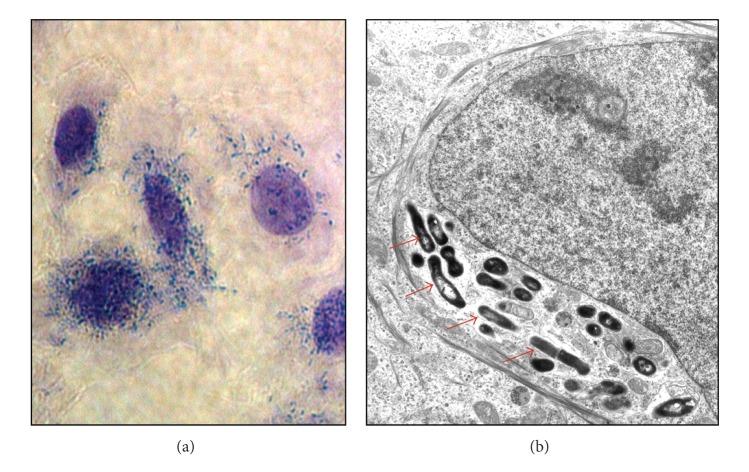
Invasiveness of *P. acnes* into epithelial cells. HEK293T cells infected with one of the serotype 1 *P. acnes* strains isolated from sarcoid lymph nodes were Giemsa stained (a) and further examined by electron microscopy (b). The electron micrographs of the cells infected with an invasive isolate show intracellular localization of the bacterium (indicated by the red arrows).

Studies of cell invading *P. acnes* are essential for linking this indigenous bacterium to the cause of sarcoidosis because infectious granulomas are commonly caused by intracellular pathogens. Furukawa et al. examined the cell invasiveness and serotype of *P. acnes* isolates from lymph nodes affected by sarcoidosis, together with isolates from nonsarcoid tissue obtained from the lymph nodes, lungs, prostate, skin, conjunctiva, and intestine [19]. The invasiveness of these *P. acnes* isolates into HEK293T cells was examined by cell invasion assay according to the method described by Cue and Cleary [20] and intracellular localization of the invasive isolates was confirmed by electron microscopy (Figure 1). Cell invasiveness was found in 14 (40%) of 35 sarcoid isolates and 65 (51%) of 127 nonsarcoid isolates. The proportion of invasive isolates did not differ between isolates from sarcoid and nonsarcoid tissues. The whole-bacterial enzyme-linked immunosorbent assays with serotype-specific antibodies discriminated the serotype of all 162 isolates (112 strains of serotype I and 50 strains of serotype II). The proportion of the two serotypes did not differ between sarcoid and nonsarcoid tissues. Cell invasiveness was found in 79 (71%) of 112 serotype I isolates and in none of 50 serotype II isolates.

## 3. Polymerase Chain Reaction

Some investigators in Europe using PCR assays detected mycobacterial DNA in samples of affected tissue from patients with sarcoidosis [21–23], but others did not [24–26]. Quantification of the bacterial genomes detected in sarcoid lesions is essential for clarifying the etiologic correlation between lesions and bacteria detected therein because a tiny volume of bacteria or bacterial DNA can be detected even in conditions of latent infection or contamination with no etiologic correlation.

**Figure 2:**
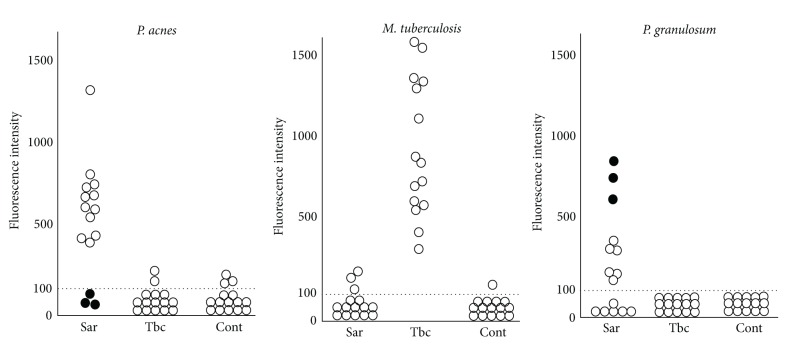
Quantitative PCR of bacterial DNA in lymph nodes from patients with sarcoidosis (Sar), tuberculosis (Tbc), and gastric cancer (Cont). The horizontal dotted lines show the detection threshold, and samples with results under this line were considered negative. Biopsy samples from the three patients with sarcoidosis but without *P. acnes* (as indicated by black dotes) all contained many *P. granulosum*.

Ishige et al. used quantitative PCR to search for bacterial genomes of *P. acnes*, *P. granulosum*, and *M. tuberculosis* in histologic sections of lymph nodes from patients with sarcoidosis, tuberculosis, or gastric cancer [27]. They examined lymph node biopsy samples from 15 patients with sarcoidosis and 15 patients with tuberculosis lymphadenitis. As controls, they examined 15 lymph nodes without metastasis from 15 patients with gastric cancer undergoing surgery (Figure 2). Genomes of *M. tuberculosis* were found in samples from all 15 patients with tuberculosis, 3 patients with sarcoidosis, and 1 control sample. Genomes of *P. acnes* were found in 12 of 15 patients with sarcoidosis, 2 tuberculosis patients, and 3 controls. The difference in the estimated number of *P. acnes* genomes between individuals with and without sarcoidosis was similar to that in the number of *M. tuberculosis* between people with and without tuberculosis. Biopsy samples from the three patients with sarcoidosis but without *P. acnes* all contained many *P. granulosum* DNA. These findings suggest that propionibacteria resided in or proliferated ectopically in the sarcoid lesions, whether or not there was a connection with the disease. Propionibacteria are more likely than mycobacteria to cause sarcoidosis.

**Figure 3:**
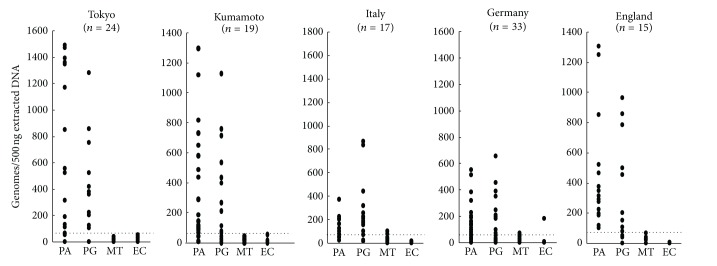
Quantitative real-time PCR of bacterial DNA in lymph node samples from Japanese and European patients with sarcoidosis. The horizontal dotted lines show the detection threshold, and samples with results under this line were considered negative. PA: *P. acnes*, PG: *P. granulosum*, MT: *M. tuberculosis*, and EC: *E. coli*.

The international collaborative study evaluated the possible etiologic link between sarcoidosis and the suspected bacterial species [8]. Formalin-fixed and paraffin-embedded sections of biopsy samples of lymph nodes, 1 from each of 108 patients with sarcoidosis and 65 patients with tuberculosis, together with 86 control samples, were collected from 2 institutes in Japan and 3 institutes in Italy, Germany, and England (Figure 3). Genomes of *P. acnes*, *P. granulosum*, *M. tuberculosis*, *M. avium subsp*. *paratuberculosis*, and *Escherichia coli* (as the control) were estimated by quantitative real-time PCR. Either *P. acnes* or *P. granulosum* was found in all but two of the sarcoid samples. *M. avium subsp*. *paratuberculosis* was not found in any sarcoid sample. *M. tuberculosis* was found in only 0% to 9% of the sarcoid samples, but in 65% to 100% of the tuberculosis samples. In sarcoid lymph nodes, the total numbers of genomes of *P. acnes* or *P. granulosum* far exceeded those of *M. tuberculosis*. *P. acnes* or *P. granulosum* was found in 0% to 60% of the tuberculosis and control samples, but the total numbers of genomes of *P. acnes* or *P. granulosum* in these samples were lower than those found in sarcoid samples. *Propionibacteriium spp*. are more likely than *Mycobacteriium spp*. to be involved in the etiology of sarcoidosis, not only in Japanese but also in the European patients with sarcoidosis.

## 4. *In Situ *Hybridization

**Figure 4:**
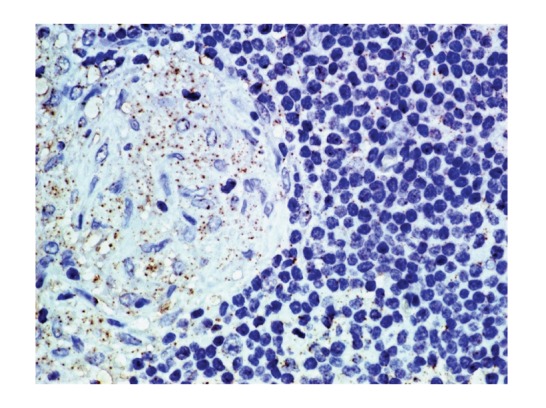
*In situ* hybridization using catalyzed reporter deposition for signal amplification with digoxigenin-labeled oligonucleotide probes that complemented 16S rRNA of *P. acnes*. Many signals were detected in the cytoplasm of sarcoid granuloma cells.

*In situ* localization of *P. acnes* genomes in sarcoid lymph nodes may help to elucidate an etiologic link between sarcoidosis and this indigenous bacterium. Formalin-fixed and paraffin-embedded biopsy samples of lymph nodes from nine patients with sarcoidosis, nine patients with tuberculosis, and nine patients with nonspecific lymphadenitis as controls were examined by quantitative real-time PCR (QPCR) for *P. acnes* and by *in situ* hybridization (ISH) using catalyzed reporter deposition (CARD) for signal amplification with digoxigenin-labeled oligonucleotide probes that complemented 16S rRNA of *P. acnes* [28]. The signals per 250 *μ*m^2^ of tissue sections from inside and outside sarcoidosis and tuberculosis granulomas and from control lymph nodes were counted. The number of genomes determined by QPCR was examined for correlation with the mean signal count by ISH with CARD. In sarcoid samples, one or several signals were detected in the cytoplasm of some epithelioid cells in granulomas (Figure 4). The mean signal counts were higher in granulomatous areas than in other areas of sarcoid lymph nodes. The correlation between the QPCR and ISH with CARD results was significant (*r* = 0.86, *P* < 0.001). The accumulation of *P. acnes* genomes in and around sarcoid granulomas suggests that this indigenous bacterium is related to the cause of granulomatous inflammation in sarcoidosis.

## 5. Immunohistochemistry

Granulomatous reactions are basically a defense mechanism that the body uses to fight off poorly degradable antigens. granulomas begin as a small collection of lymphocytes and macrophages surrounding poorly degradable antigens. The aggregating macrophages, called an early focus of granuloma, then change to epithelioid cells and become organized into a cluster of cells, called an immature granuloma. Further progression results in ball-like clusters of cells and fusion of macrophages into giant cells, called a mature granuloma. The questions that must be asked in searching for the cause of sarcoidosis, therefore, are: “What is the antigen that the granulomas are fighting?” and “How is the antigen localized within the sarcoid lesion?” To evaluate the pathogenic role of *P. acnes*, Negi et al. screened for this indigenous bacterium in sarcoid and nonsarcoid tissues using immunohistochemical methods with novel *P. acnes*-specific monoclonal antibodies that react with cell-membrane-bound lipoteichoic acid (PAB antibody) and ribosome-bound trigger factor protein (TIG antibody). They examined formalin-fixed and paraffin-embedded samples of lungs and lymph nodes from 196 patients with sarcoidosis and corresponding control samples from 275 patients with nonsarcoidosis diseases. The samples were mostly from Japanese patients, with 64 lymph node samples from German patients [29].

**Figure 5:**
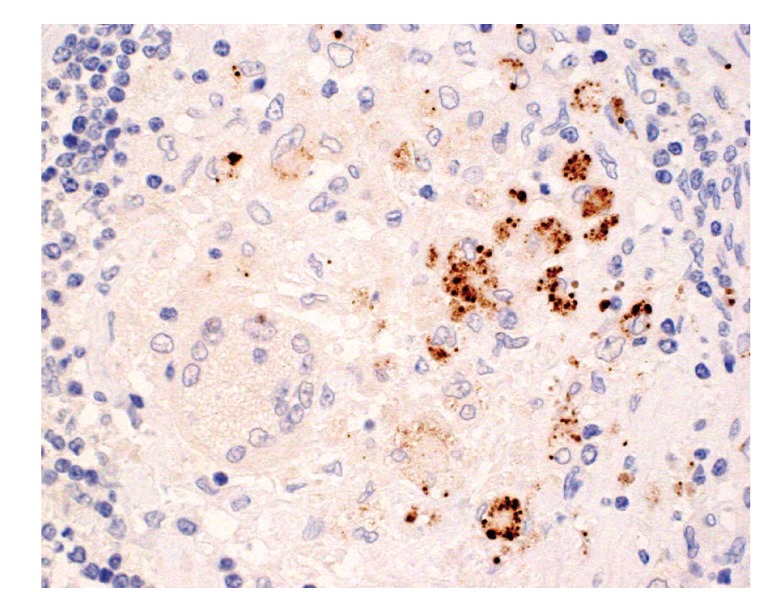
Immunohistochemistry with a *P. acnes*-specific monoclonal antibody (PAB antibody) that reacts with cell-membrane-bound lipoteichoic acid of the bacterium. Many small round bodies are shown within a noncaseating epithelioid cell granuloma of sarcoid lymph node.

**Figure 6:**
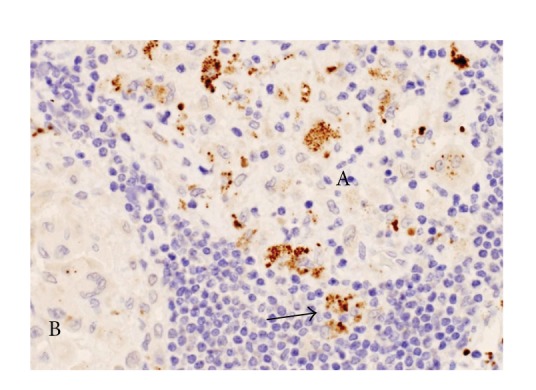
Many small round bodies detected by the PAB antibody are shown intermingled with many lymphocytes in one immature granuloma (A), but only a few are observed in the mature granuloma (B) of the sarcoid lymph node. Most of the *P. acnes* are present within the granuloma, but some are present outside of the granuloma (as indicated by the arrow).

**Figure 7:**
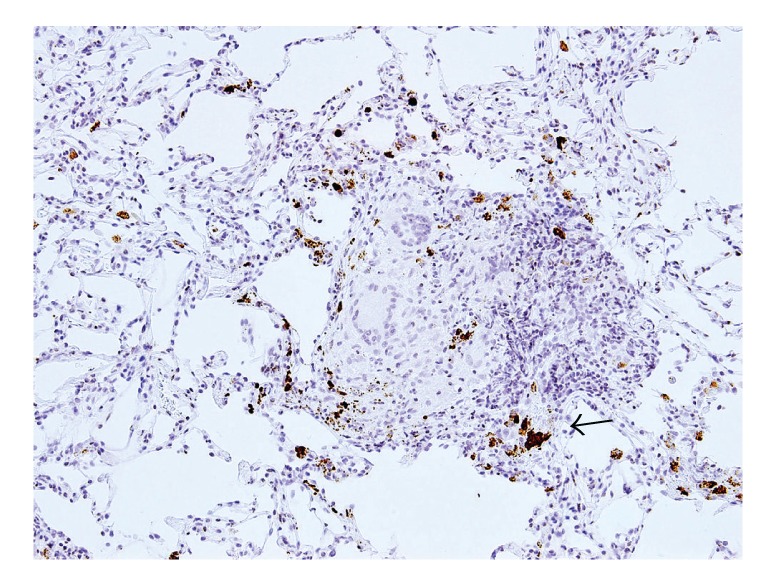
In the lung sarcoid granuloma lesion surrounded by prominent inflammatory cell infiltration, small round bodies are detected by the PAB antibody not only in the granuloma cells but also in some of the inflammatory cells. The arrow indicates the magnified region shown in Figure 8.

**Figure 8:**
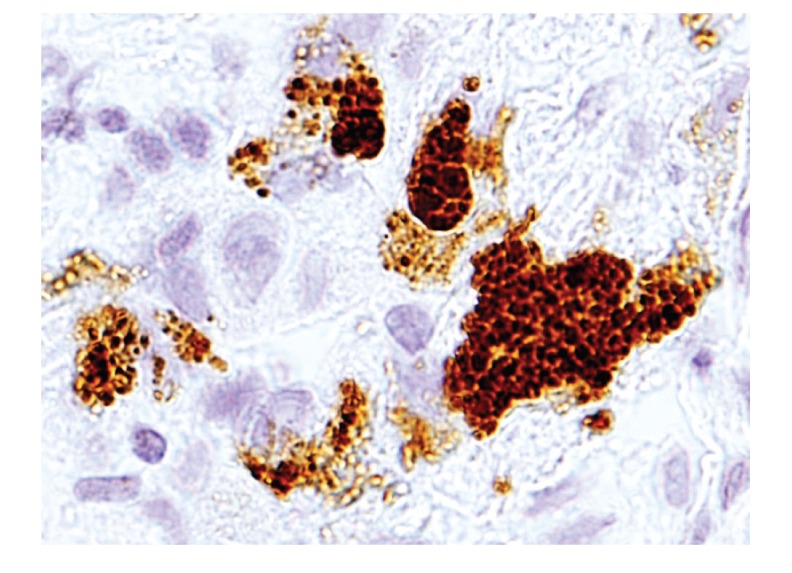
Higher magnification of the area indicated by the arrow in Figure 7. Some swollen macrophages of the immature granuloma are filled with many small round bodies detected by the PAB antibody.

Immunohistochemistry with the PAB antibody revealed small round bodies within sarcoid granulomas in 20/27 (74%) video-assisted thoracic surgery lung samples, 24/50 (48%) transbronchial lung biopsy samples, 71/81 (88%) Japanese lymph node samples, and 34/38 (89%) German lymph node samples. The PAB antibody did not react with nonsarcoid granulomas in any of the 45 tuberculosis samples or the 34 samples with sarcoid reaction. The appearance of the small round bodies detected by the PAB antibody within sarcoid granulomas did not differ between lungs and lymph nodes. In sarcoid granulomas with many small round bodies, the cytoplasm of some granuloma cells was filled with small round bodies, consistent with the intracellular proliferation of the bacterium (Figure 5). In many sarcoid granulomas, a few small round bodies with occasional degraded or large-sized features were scattered among the granuloma cells. The amount of these small round bodies varied from each granuloma in identical sarcoid samples as well as from each sarcoid tissue sample (Figure 6). The appearance of the small round bodies detected by the PAB antibody within sarcoid granulomas did not differ between lungs and lymph nodes (Figures 7 and 8).

**Figure 9:**
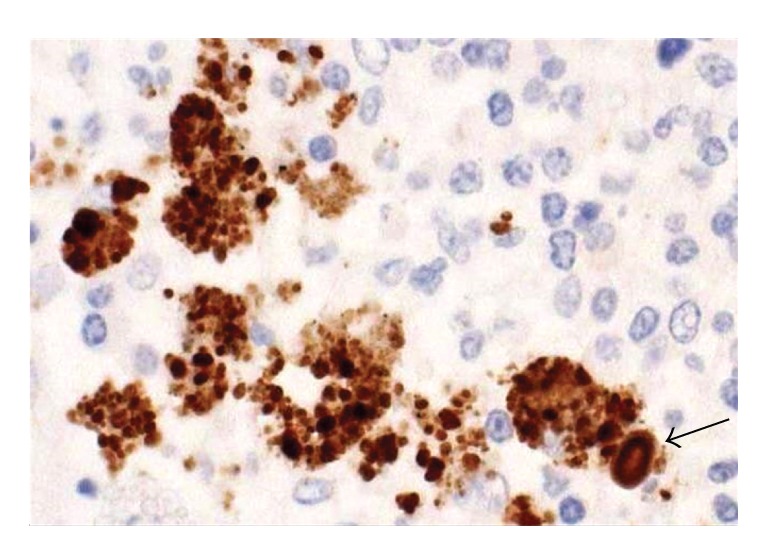
A cluster of some swollen macrophages filled with many small round bodies detected by the PAB antibody is occasionally found in paracortical areas of sarcoid lymph nodes. The arrow indicates a large-spheroidal body similar to Hamazaki-Wesenberg bodies.

**Figure 10:**
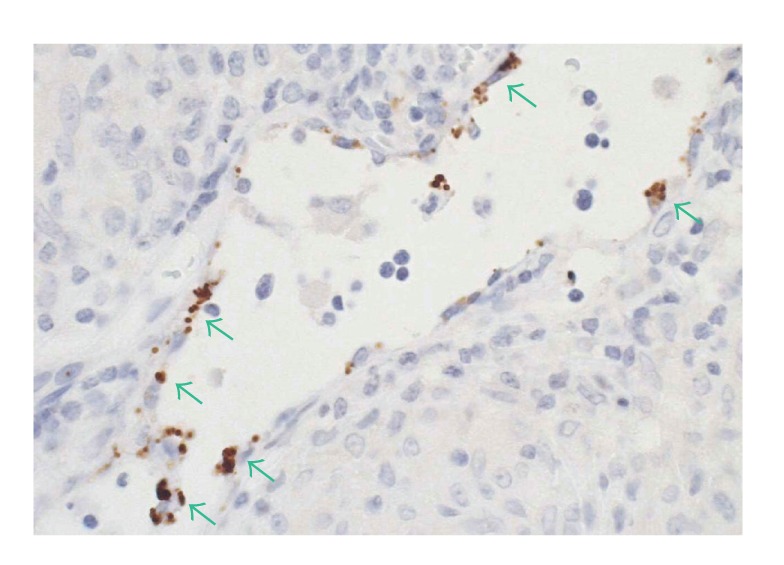
Some of the small round bodies detected by the PAB antibody (green arrows) were observed in lymphatic endothelial cells adjacent to sarcoid granulomas of the lymph node.

In nongranulomatous areas small round bodies detected by the PAB antibody were found in alveolar macrophages of lungs and paracortical macrophages of lymph nodes from many sarcoid and some nonsarcoid patients. In the lymph nodes, paracortical macrophages with many small round bodies detected by the PAB antibody (Figure 9) were observed in 26 (22%) of 119 sarcoid samples and 18 (11%) of 165 nonsarcoid samples. The frequency was significantly higher in the sarcoid samples. Such small round bodies were observed in lymphatic endothelial cells in a few samples of sarcoid lymph nodes (Figure 10). In the lungs, alveolar macrophages with many small round bodies detected by the PAB antibody were found in 28 (36%) of 77 sarcoid samples and 18 (16%) of 110 nonsarcoid samples. The frequency was significantly higher in sarcoid samples. Such alveolar macrophages occasionally contained one or a few large spheroidal bodies detected by the PAB antibody that were acid-fast with Fite staining and also reacted with the TIG antibody.

**Figure 11:**
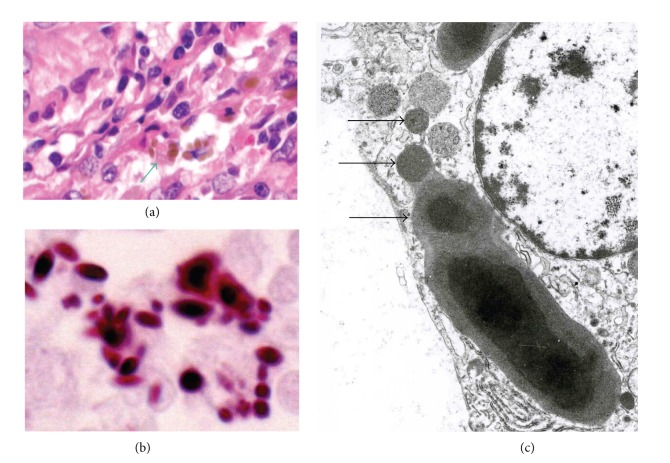
Hamazaki-Wesenberg (HW) bodies in sarcoid lymph nodes. HW bodies are large and spheroidal in shape with a yellow-brown color, as indicated by the green arrow, with hematoxylin and eosin staining (a). These bodies are strongly acid-fast with Fite staining (b). HW bodies with one-by-one protrusions (c), as indicated by black arrows, are rarely found in sinus macrophages of sarcoid lymph nodes.

**Figure 12:**
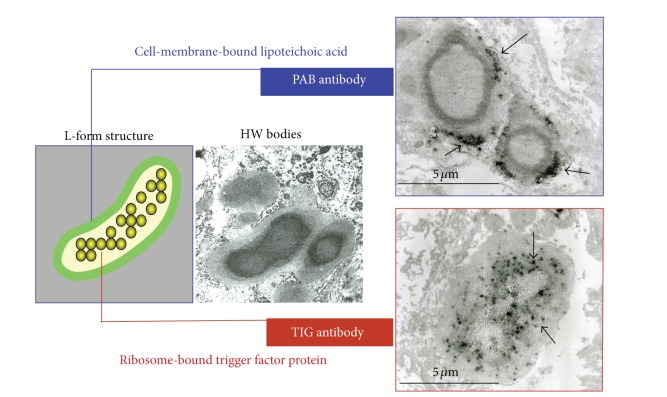
Immunoelectron-microscopic analysis with PAB and TIG antibodies suggests that HW bodies may be cell-wall-deficient *P. acnes*. Representative immunoreactive products of the antibodies are indicated by black arrows.

Hamazaki-Wesenberg (HW) bodies frequently appear in sarcoid lymph nodes although these bodies are not specific to sarcoidosis [30–32]. The large-spheroidal acid-fast bodies, HW bodies, which were found in 50% of sarcoid and 15% of nonsarcoid lymph node samples, reacted with both PAB and TIG antibodies. Electron microscopy revealed that these HW bodies had a single bacterial structure and lacked a cell wall with occasional protrusions from the body (Figure 11). Immunoelectron microscopy revealed that the immunoreactive products of the PAB antibody and TIG antibody were differentially distributed in the outer and inner areas of the HW bodies, respectively (Figure 12). The localization of cell-membrane-bound lipoteichoic acid detected by the PAB antibody and ribosome-bound trigger factor detected by the TIG antibody suggests that HW might not be phagolysosomally-degraded products of *P. acnes*, but rather intact forms of intracellular bacteria because the original distribution pattern (plasmalemmal and protoplasmic localization, resp.) of these bacterial components was preserved in terms of the morphologic structure of the bacterium. Furthermore, conventional electron microscopy revealed that these bodies lack a cell-wall structure and occasionally exhibit protrusions from the body that appear to be yeast-like proliferating features (not mitotic, but sprouting or branching), characteristic of cell-wall-deficient (L-form) bacteria (Figure 11). HW bodies may be cell-wall-deficient *P. acnes*.

**Figure 13:**
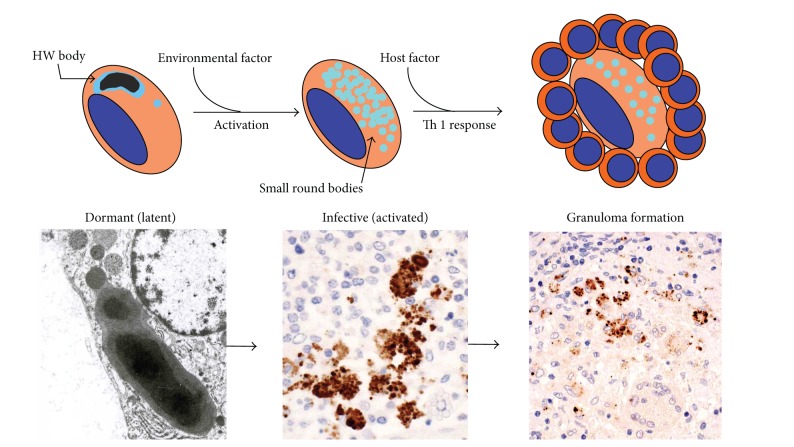
Hypothesized mechanism of sarcoid granuloma formation caused by *P. acnes*. Intracellular proliferation of *P. acnes* in macrophages triggers granuloma formation in patients with hypersensitivity to this indigenous bacterium.

Histopathological analysis with the PAB antibody led us to formulate a hypothesis for the mechanism of granuloma formation in sarcoidosis (Figure 13). *P. acnes* causes latent infection and persists in macrophages. HW bodies are dormant and cell-wall-deficient *P. acnes*. This dormant form of *P. acnes* can be activated endogenously under certain environmental conditions and proliferate in cells at the sites of latent infection. Small round bodies proliferating in macrophages are infective forms of *P. acnes*. When these bodies spread out of macrophages, they infect other cells or organs via the lymph and blood streams. Sarcoid granulomas are formed as a host defense mechanism at the sites of activated bacteria proliferating intracellularly in patients with hypersensitive immune responses to *P. acnes* to prevent the spread of the infectious agent.

**Figure 14:**
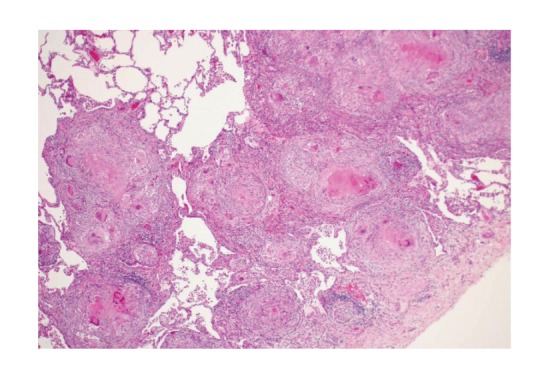
A lung sample with many epithelioid cell granulomas with central eosinophilic necrosis. This case required differential diagnosis for *M. tuberculosis* although the specimen contained no acid-fast bacilli and the clinical data of the patient suggested sarcoidosis.

**Figure 15:**
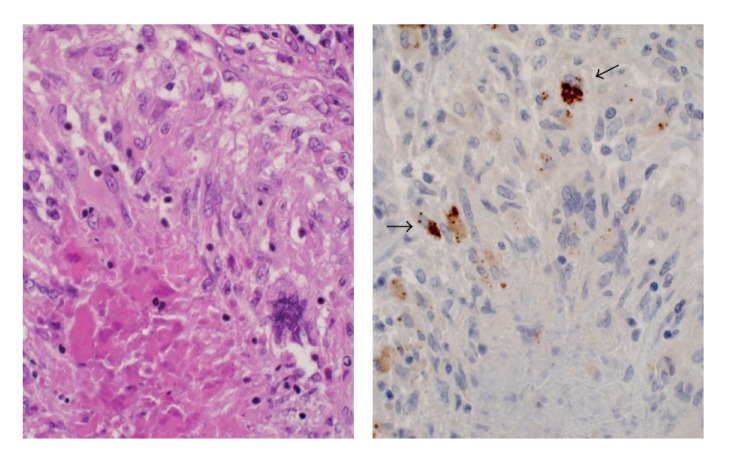
Immunohistochemistry with the PAB antibody for the specimen shown in Figure 14 revealed positive reaction products (black arrows) within granulomas accompanied by central eosinophilic necrosis.

The PAB antibody seems to be appropriate for detecting cell-wall-deficient *P. acnes* because an epitope of lipoteichoic acid detected by this antibody is more exposed in cell-wall-deficient forms than in conventional forms of the bacterium. The high frequency and specificity of *P. acnes* detected by the PAB antibody within sarcoid granulomas suggest an etiologic link between sarcoidosis and this indigenous bacterium. The PAB antibody may be useful for diagnosing sarcoidosis caused by *P. acnes*, when the reactivity is detected in idiopathic granulomas (Figures 14 and 15). The TIG antibody seems to be appropriate for detecting latent forms of *P. acnes* because increased expression of the trigger factor protein is found only in the HW bodies. The trigger factor protein is a molecular chaperone, like some heat-shock proteins, and either overproduction or depletion of the trigger factor protein causes filamentation indicative of cell division defects. Increased expression of the trigger factor protein in HW bodies might be necessary to sustain the latent phase of intracellular persistent bacterium.

## 6. Host Factor

Host factors may be more critical than agent factors in the etiology of sarcoidosis, as suggested by the Kveim test phenomenon [33], in which an intracutaneously injected suspension of sarcoid tissue causes sarcoid granulomas in patients with sarcoidosis but not in healthy people or patients with other diseases. The inflammatory response in sarcoidosis involves many activated T cells and macrophages [34], with a pattern of cytokine production in the lungs consistent with a helper T-cell type 1 (Th1) immune response triggered by undefined antigen(s) [35]. If a *propionibacterium* caused a particular case of sarcoidosis, it is likely that an antigen arising from the bacterium gave rise to a Th1 immune response in the subject.

**Figure 16:**
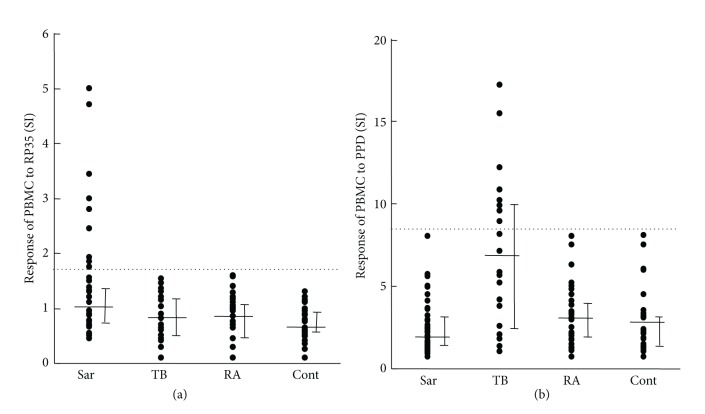
Response of peripheral blood mononuclear cells (PBMCs) to recombinant trigger factor protein (RP35) from *P. acnes* and purified protein derivative (PPD) from *M. tuberculosis*. The horizontal bars show, from bottom to top, the 25th percentile, median, and 75th percentile, respectively. The dotted lines show the threshold, set at the mean + 3SD of the 32 control samples. Sar: Sarcoidosis (*n* = 50), TB: tuberculosis (*n* = 21), RA: rheumatoid arthritis (*n* = 32), and Cont: healthy volunteer (*n* =32).

Ebe and colleagues searched for propionibacterial antigens that evoked cellular immune responses only in patients with sarcoidosis [11]. For this purpose, a *λ*gt11 genomic DNA library of *P. acnes* was screened with sera from patients with sarcoidosis, because high levels of serum antibodies against the antigen usually accompany such an immune response. Of 180,000 plaques screened, 2 clones coded for an identical recombinant protein, RP35, recognized by sera. RP35, a recombinant protein of 256 amino acid residues with a calculated molecular mass of 28,133 Da, is a fragment (the C-terminal region) of the *P. acnes* trigger factor, which has 529 amino acid residues and a calculated molecular mass of 57,614 Da. The C-terminal sequence (Asp-463 to Lys-529) seems to be unique to *P. acnes*, with no similarity to sequences of other bacterial proteins deposited in the Swiss-Prot database. Conformational analysis of the Ser-491 to Lys-529 regions at the C terminus revealed it to be highly antigenic. RP35 caused sarcoidosis-specific proliferation of peripheral blood mononuclear cells (PBMCs) from 9 (18%) of 50 patients with sarcoidosis (Figure 16). The same study established that serum levels of IgG and IgA antibodies to RP35 are high in patients with sarcoidosis and other lung diseases. In bronchoalveolar lavage (BAL) fluid, IgG and IgA antibody levels were high in 7 (18%) and 15 (39%), respectively, of 38 patients with sarcoidosis, and in 2 (3%) and 2 (3%), respectively, of 63 patients with other lung diseases. The results of the study suggested that this antigen from *P. acnes* is responsible for the formation or maintenance of granulomas in some patients with sarcoidosis.

Recently, Furusawa et al. [36] reported that interleukin-2 secretion from PBMCs after stimulation with viable *P. acnes* is higher in patients with sarcoidosis than in control subjects. Interleukin-2 and interleukin-12 mRNA expression of PBMCs after stimulation with *P. acnes* is also higher in patients with sarcoidosis than in control subjects. In contrast, interleukin-17 mRNA expression of PBMCs is lower in patients with sarcoidosis than in control subjects. The responses of the two groups to the stimulation with *M. tuberculosis* antigens such as Bacille de Calmette et Guérin (BCG) or ESAT-6 recombinant protein were not significantly different. Sarcoidosis may arise from an imbalance of Th1/Th17 immune responses to viable *P. acnes*.

Additional evidence of the hypersensitivity of sarcoid patients to *P. acnes* was obtained in studies of BAL cells. When stimulated with a crude extract of *P. acnes* with pyridine, BAL cells from patients with sarcoidosis proliferated more than BAL cells from healthy subjects or from patients with lung cancer [37]. Interleukin-2 production and interleukin-2 receptor expression of BAL cells stimulated by the *P. acnes* antigen was greater in sarcoidosis patients than in healthy subjects or patients with other lung diseases [38]. *P. acnes* DNA was detected in BAL cells from 21 (70%) of 30 sarcoid patients and 7 (23%) of 30 control patients with other lung diseases [39]. *In situ* signals of *P. acnes* DNA were detected in the cytoplasm of a few alveolar macrophages among the BAL cells from sarcoid patients, but from no other kinds of BAL cells, including alveolar lymphocytes and neutrophils. Gallium-67 uptake by lung parenchyma was found in about half of the 30 sarcoid patients with *P. acnes* DNA, but in none of the other sarcoid patients [39]. 

**Figure 17:**
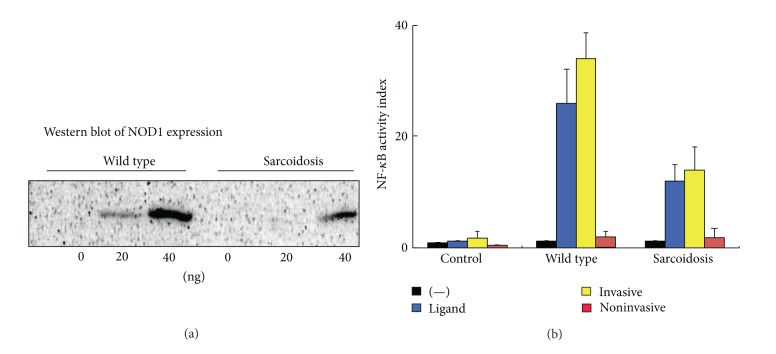
Functional studies (b) revealed that intracellular *P. acnes* activates NF-*κ*B in a NOD1-dependent manner and the NOD1 796A-allele predominant in sarcoidosis patients causes diminished NF-*κ*B activation in response to intracellular *P. acnes*. Western blot analysis (a) shows reduced expression of the NOD1 796A-allele.

Mutations in the related NOD2 gene predispose patients to granulomatous diseases, including Crohn's disease [40], Blau syndrome [41], and early-onset sarcoidosis [42]. Although Blau syndrome and early-onset sarcoidosis are reported to share identical NOD2 mutations, no association has been reported between NOD2 and sarcoidosis [43]. NOD1 shares many structural and functional similarities with NOD2. Tanabe et al. found that intracellular *P. acnes* activates NF-*κ*B in both an NOD1- and NOD2-dependent manner [44]. A systematic search for NOD1 gene polymorphisms in Japanese sarcoidosis patients identified two alleles, 796G-haplotype (156C, 483C, 796G, and 1722G) and 796A-haplotype (156G, 483T, 796A, and 1722A). Allelic discrimination of 73 sarcoidosis patients and 215 healthy individuals showed that the frequency of the 796A-type allele is significantly higher in sarcoidosis patients and the odds ratios (ORs) are significantly elevated in NOD1-796G/A and 796A/A genotypes (OR (95% CI) = 2.250 (1.084, 4.670) and 3.243 (1.402, 7.502), resp.) as compared to the G/G genotype, showing an increasing trend across the 3 genotypes (*P* = 0.006 for trend). Functional studies indicated that the NOD1 796A-allele is associated with reduced expression leading to diminished NF-*κ*B activation in response to intracellular *P. acnes* (Figure 17).

*P. acnes* has been studied for its role in immunomodulation with the conclusion that Toll-like receptor 2 (TLR2), TLR4, and TLR9 mediate the effects of *P. acnes* infection [45, 46]. These studies, however, only investigated noninvasive *P. acnes*. TLRs are likely to serve as first-line receptors for *P. acnes*, but NOD proteins might play a major role in a subsequent phase of intracellular infection. NOD1 was recently reported to be a critical regulator of beta-defensin 2 during Helicobacter pylori infection [47]. It is possible that impaired expression of beta-defensin 2 through 796-A NOD1 due to a reduced ability to induce NF-*κ*B enables *P. acnes* to survive and persist intracellularly, leading to the pathogenesis of sarcoidosis.

## 7. Experimental Models

In experimental animals, granulomatous lesions can be induced by *P. acnes*. A single intravenous injection of *P. acnes* into mice leads to the development of many granulomas in the liver [48–50], but not in the lungs. Pulmonary granulomas can be induced, however, by an intravenous injection of *P. acnes* into sensitized rats [51] and rabbits [52]. In these two studies of experimental pulmonary granulomas, heat-killed *P. acnes* was used as a sensitizer, and a challenge by a single intravenous injection of the bacterium was essential for granulomas to form in the lungs.

**Figure 18:**
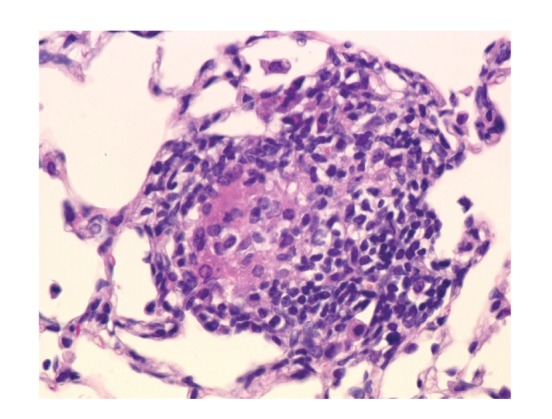
A noncaseating epithelioid cell granuloma observed in a mouse with experimental pulmonary granulomatosis induced by sensitization with *P. acnes* trigger factor protein and adjuvant.

*P. acnes* trigger factor protein (RP35) or heat-killed *P. acnes* causes pulmonary granulomas in some (25%–57%) mice sensitized with the protein and complete Freund's adjuvant (CFA) [53]. An intravenous injection of *P. acnes* as a challenge was not essential for granulomas to form in the lungs. Granulomas were scattered throughout the lungs, especially in subpleural areas (Figure 18). The granulomas were composed of a core of epithelioid cells intermingled with a few and surrounded by many mononuclear cells. The detection frequency of pulmonary granulomas did not differ significantly between mice sensitized with the RP35 or heat-killed *P. acnes*. 

This experimental protocol may provide a satisfactory model of sarcoidosis. First, hypersensitivity to *P. acnes* trigger factor, such as has been experimentally induced, has been reported in some patients with sarcoidosis. Second, situations resembling intravenous challenge with *P. acnes* are rare in humans, and sarcoidosis can start in asymptomatic persons without evidence of septicemia.

Experimental models of allergic diseases, such as encephalomyelitis [54], thyroiditis [55], and orchitis [56], have been produced by immunizing animals with self-antigens (myelin basic protein, thyroglobulin, and testicular homogenate, resp.) emulsified in CFA, which is essential for the experiment. Autoimmune inflammatory lesions are induced in this way only in the organs from which the self-antigens used for the sensitization originated. In the animal model of sarcoidosis, sensitization of mice with *P. acnes* trigger factor protein or heat-killed *P. acnes* in CFA induces granulomatous inflammation confined to the lungs. This finding suggests that such antigens from *P. acnes* exist in the lungs of mice even before the experiment. 

Similar to the results obtained by bacterial culture of human samples from lung and lymph nodes, *P. acnes* was cultured from the lungs, liver, and lymph nodes from some of the untreated normal mice, and culture was most often successful with the lungs. There was an unexpected concordance in the rate (33%) of culture from normal lungs and the frequency of detection of pulmonary granulomas in mice sensitized with the trigger factor protein. The concordance suggests that mice without granulomas may have been free from *P. acnes* in the normal indigenous flora of their lungs before and during the experiment. 

**Figure 19:**
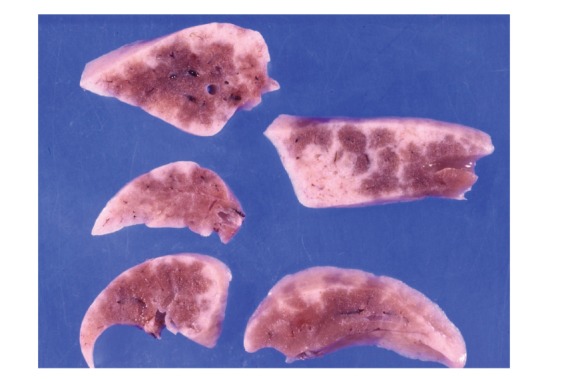
Cut sections of the lungs from a rabbit with experimental pulmonary granulomatosis induced by sensitization with *P. acnes* trigger factor protein and adjuvant. Whitish lesions are distributed throughout and are especially prominent in the subpleural and interlobular areas.

**Figure 20:**
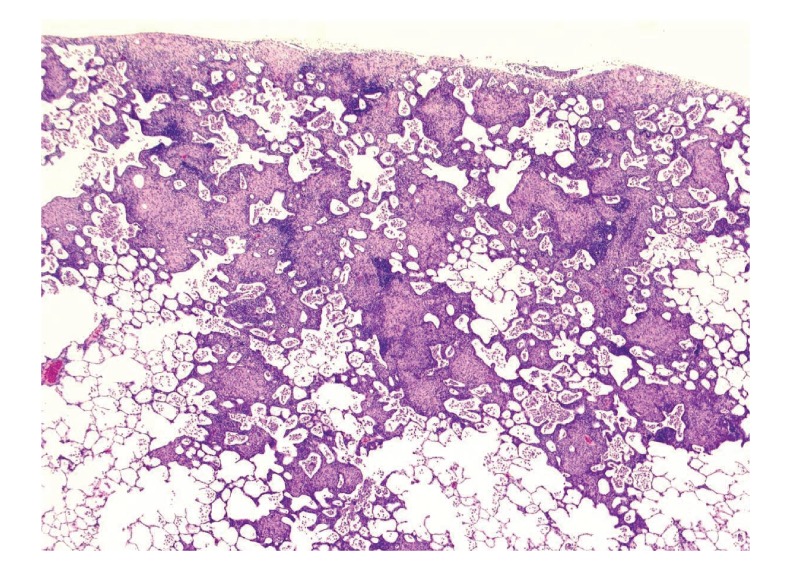
Histologic features of experimental pulmonary granulomatosis of the rabbit shown in Figure 19. Multiple noncaseating epithelioid cell granulomas are accompanied by surrounding lymphoid cell infiltration with alveolitis.

**Figure 21:**
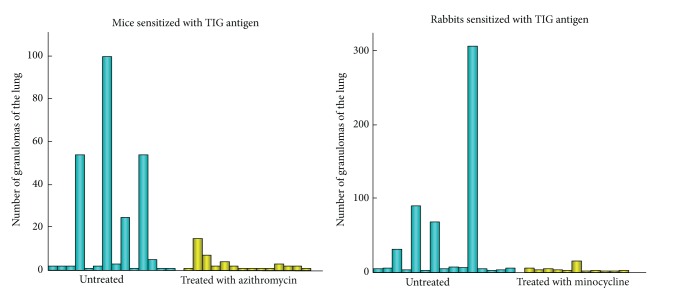
Prevention of pulmonary granulomatosis in mice and rabbits sensitized with *P. acnes* trigger factor (TIG) antigen by administration of antibiotics before and during the experiments.

Using the same experimental protocol with rabbits bred in a conventional environment, rabbits sensitized with the *P. acnes* trigger factor antigen developed more severe and diffuse pulmonary granulomatosis than did sensitized mice. In fact, the severe granulomatous inflammation that developed could be even identified macroscopically (Figures 19 and 20). Th1 immune response to the *P. acnes* trigger factor protein might have caused pulmonary granulomas at the sites of *P. acnes* infection. Indeed, the administration of antibiotics (azithromycin in mice and minocycline in rabbits) before and during the experiments prevented granuloma formation in these experimental models (Figure 21).

Nishiwaki et al. further examined the mechanism of pulmonary granulomatosis caused by the sensitization of heat-killed *P. acnes* [57], using a similar experimental protocol as used in a previous study [53]. In the study, *P. acnes* was identified in normal murine alveolar cells by immunostaining with a *P. acnes*-specific monoclonal antibody (PAB antibody). *P. acnes* was taken up by lung cells, and the *P. acnes*-bearing cells expressed F4/80 rather than CD11c or DEC205, consistent with the ability of macrophages to phagocytose and deliver antigens to dendritic cells in the lung. As far away as the end of the airway, airborne organisms are impacted and eliminated by mechanical defenses, including mucociliary clearance and coughing. Nevertheless, a small number of *P. acnes* might escape this system and reside on the alveolar surface. *P. acnes* genomes were detected in normal pulmonary lymph nodes as well as the lungs, and lymphocytes from lymph nodes showed *P. acnes*-specific proliferation, suggesting that these cells had already been exposed to *P. acnes* by lung-derived antigen-presenting-cells and had established a memory response. Additionally, these results indicate that *P. acnes* were continuously transported to pulmonary regional lymph nodes in the steady state. Because of this constant delivery of antigens to the pulmonary lymph nodes for a long period, the small number of indigenous *P. acnes* in the normal lung would be enough to produce a specific immune response, but not for the formation of a steady-state granuloma. Although mycobacterial, atypical mycobacterial, and other propionibacterial antigens are potential candidate endogenous microorganisms that trigger pulmonary granuloma formation, genomic analyses revealed an absence of these organisms in the lungs of specific-pathogen-free C57BL/6 mice. 

The adoptive transfer of *P. acnes*-sensitized lymph node CD4+ T cells into naïve mice resulted in granulomatous changes in the lung, indicating that extrapulmonary lymph node CD4+ T cells primed with *P. acnes* can interact with pulmonary resident cells via the circulation and induce granuloma formation in the normal lung. It was therefore hypothesized that a continuous supply of *P. acnes*-sensitized T cells should lead to chronic pulmonary granuloma formation, and consequently, we performed continuous remote sensitization of normal mice with *P. acnes*. These mice exhibited distinct pulmonary granulomas, distributed in lymph-rich spaces such as the subpleural, peribronchial, and perivascular areas and had typical cellular components of granuloma and preferential Th1 cytokine expression. These features are similar to those of pulmonary sarcoidosis. In addition, the ratio of CD4 to CD8 BAL lymphocytes was elevated in the group immunized twice, and serum calcium levels were also increased. Thus, the characteristics of this *P. acnes*-immunization model, without any direct exposure of antigen to the lung, showed several similarities to those of sarcoid patients.

That study also examined whether changes in the number of preexisting *P. acnes* cells in the lung affected pulmonary granuloma formation. As expected, preloading of *P. acnes* exacerbated pulmonary disorders, whereas reduction of the *P. acnes* population by antimicrobial treatment reduced the pulmonary lesions. These findings suggest a pivotal role of normally localized *P. acnes* in the formation of pulmonary granuloma by extrapulmonary *P. acnes* sensitization, as well as the potential clinical usefulness of antimicrobial eradication targeting lung-indigenous *P. acnes* for the treatment of pulmonary granulomatosis induced by similar pathogenesis.

## 8. Etiology of Sarcoidosis

**Figure 22:**
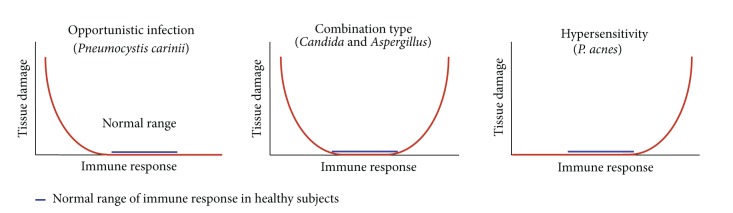
Three major categories of endogenous infection in the classification system of diseases caused by indigenous microorganisms.

In the past, once the germ theory of disease was accepted, microbes were considered to be pathogens if they met the stipulations of Koch's postulates. Although there are many microbes, however, most human infections are caused by only a few. Some microbes have been classified as pathogens although they do not cause disease in every host. In addition, some microbes have been classified as nonpathogenic although they cause disease in certain hosts. For these reasons, in a redefinition of the concepts of virulence and pathogenicity of microbes, Casadevall and Pirofski suggested a classification system for pathogens based on their ability to cause damage as a function of the host's immune response [58]. Koch's postulates for exogenous infection cannot be applied to diseases caused by endogenous bacteria. Endogenous infection is a disease caused by indigenous microorganisms. According to the classification system suggested by Casadevall and Pirofski, endogenous infection, which does not cause any lesions under normal immune conditions, can be classified into three major categories (Figure 22). Opportunistic infections, such as *Pneumocystis carinii* pneumonia, are well known to be associated with immunodeficiency in AIDS patients. Combination type infections, such as *Candida* and *Aspergillus*, not only cause opportunistic infections, but may also cause hypersensitivity pneumonitis. The hypersensitivity type of endogenous infection does not cause any tissue damage until the hypersensitive immune response is triggered. *P. acnes* as a cause of sarcoidosis can therefore be classified within the group of endogenous diseases that results from hypersensitivity.

*P. acnes* is the most [12] common commensal bacterium in the lungs and lymph nodes from subjects without sarcoidosis [18]. Some *P. acnes* is found in 20% of nonsarcoid lymph nodes by bacterial culture [18], 15% of nonsarcoid lymph nodes by PCR [27], 18% of nonsarcoid lung samples, and 22% of nonsarcoid lymph node samples by immunohistochemistry [29]. Occasional detection of intracellular *P. acnes* in nongranulomatous areas of the lungs and lymph nodes from nonsarcoid patients suggests that latent infection and endogenous reactivation of this indigenous bacterium occurs in these organs, even in patients without sarcoidosis. Sarcoidosis involves many organs, and the lungs and mediastinal lymph nodes are involved at the highest frequency [59]. Commensalism of *P. acnes* in these organs may explain why they are frequently involved in sarcoidosis.

**Figure 23:**
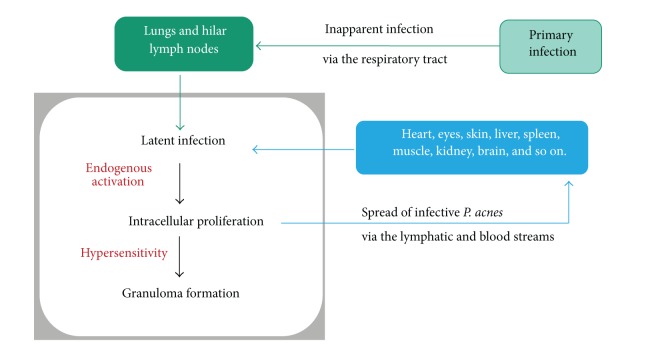
Hypothesized mechanism of systemic sarcoid granuloma formation caused by *P. acnes*.

*P. acnes*, indigenous low-virulence bacterium, can cause latent infection in the lungs and lymph nodes and persist in a cell-wall-deficient form. This dormant form of *P. acnes* can be activated endogenously under certain environmental conditions and then proliferate in cells at the site of the latent infection. In patients hypersensitive to this endogenous bacterium, granulomatous inflammation is triggered by intracellular proliferation of the bacterium. Some proliferating bacteria may escape from isolation by the granuloma and spread to other organs via the lymph and blood streams. The spread of infective *P. acnes* might cause a new latent infection in systemic organs, such as eyes, skin, and heart. Latent infection established in certain systemic organs will be reactivated simultaneously by the next triggering event, resulting in the onset of systemic sarcoidosis (Figure 23). 

Intracellular proliferation of *P. acnes* triggered by endogenous activation of latent infection might lead to the spread of the infectious *P. acnes*, giving rise to a new latent infection even within the same organ. As long as such latent infection is inadequately eradicated by the host defense mechanism of granuloma formation, the process will be repeated anytime the reactivation occurs under the requisite environmental conditions. Relapsing sarcoidosis causes repetitive acute inflammation and postinflammatory scars in the affected organs, which results in the progression of sarcoidosis through tissue damage and functional disorder in the affected organ. 

Sarcoidosis is most likely the result of a complex interaction between infection, immunity, and allergic reaction. There are three conditions essential to the development of sarcoidosis caused by *P. acnes*: (1) latent infection with cell-wall-deficient *P. acnes*, (2) endogenous activation of dormant *P. acnes* triggered by certain environmental factors, and (3) a hypersensitive Th1 immune response towards the intracellular proliferation of *P. acnes*. The formation of sarcoid granulomas might be induced by a Th1 immune response to one or more antigens of *P. acnes* proliferating in the affected organ or tissue in an individual with a hereditary or acquired abnormality of the immune system. 

Tuberculosis shares many common features with sarcoidosis, not only their histopathological features, but also aspects of their pathogenesis. Many tuberculosis cases arise from the endogenous activation of latent tuberculosis infection. Primary *M. tuberculosis* infection usually occurs in childhood and produces lesions termed the “primary complex,” which is a combination of lesions in the lung and lung hilar lymph nodes. Around 90% of subjects with primary infection by *M. tuberculosis* exhibit a so-called “unapparent infection;” that is, they are asymptomatic. Latent infection by this pathogen is characterized by healed lesions comprising consolidated scar tissue or necrotic lesions that often becomes calcified. The persistent mycobacteria in this dormant phase are thought to be cell-wall-deficient. Active tuberculosis occurs when the latent mycobacterial infection is endogenously activated under certain environmental conditions, especially in older people. The risk for activation is also significantly increased by immunosuppressive triggers, such as HIV infection and diabetes. Recent immunologic data provide evidence of latent tuberculosis in about one-third of the global population, which corresponds to more than 2 billion individuals [60].

Endogenous reactivation of latent bacteria is well known to occur in tuberculosis, which shares many common features with sarcoidosis, not only the histopathological features, but also the pathogenic features. Many cases of adult tuberculosis are caused by endogenous activation of latent tuberculosis infection [60, 61]. Tuberculosis and sarcoidosis are side effects of the antitumor necrosis factor-*α* drugs administered to patients with rheumatoid arthritis [62, 63]. Antitumor necrosis factor-*α* treatment is thought to reactivate latent tuberculosis infection [64]. In the same manner, latent *P. acnes* might be reactivated by antitumor necrosis factor-*α* treatment, resulting in sarcoidosis in certain susceptible subjects among these patients.

## 9. Treatment of Sarcoidosis

Immunosuppressive, mainly corticosteroid, therapy has been used for sarcoidosis for more than 50 years, but the long-term effects of steroidal treatment in chronic sarcoidosis are still disputed [3]. Further, the high relapse rate after treatment and the side effects of long-term use are often a clinical challenge [65]. Steroids suppress the allergic reaction, thereby providing therapeutic effects. Interference of the inflammatory process by these immunosuppressive drugs, however, may impede the formation of granulomas, which function to curtail the spread of *P. acnes* infection.

**Figure 24:**
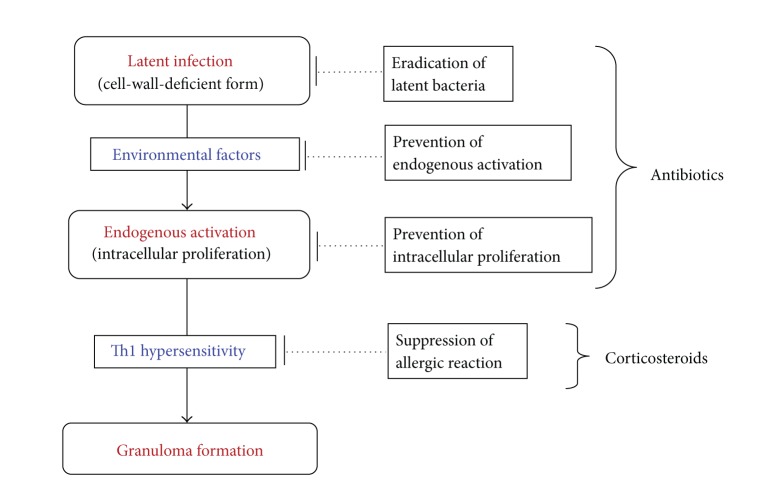
Strategy for treating sarcoidosis caused by *P. acnes*.

Antibiotics not only kill the bacteria proliferating in cells, but they also prevent the endogenous activation of latent bacteria. Long-term administration of antibiotics may therefore be effective for patients with progressive sarcoidosis by preventing inflammatory relapses caused by reactivation of the latent bacteria (Figure 24). If latent bacterial infection persisting in organs can be eradicated by treatment with antibiotics, complete remission of sarcoidosis may be achieved. Complete eradication of latent bacteria might be difficult to achieve through the conventional use of antibiotics, however, as in the case of pulmonary tuberculosis. 

Another approach for treating sarcoidosis is specific suppression of the hypersensitivity to *P. acnes* antigens responsible for granuloma formation, such as the trigger factor protein. Recent advances in the modulation of epitope-specific immune responses by synthetic peptides might help to improve the treatment of sarcoidosis. 

Minocycline is the first-choice antibiotic for patients with acne vulgaris caused by *P. acnes*. In an observational study, Bachelez and colleagues [66] reported the possible benefits of tetracyclines, to which *P. acnes* is sensitive, for the treatment of chronic forms of cutaneous sarcoidosis. The authors treated 12 patients with biopsy-proven cutaneous sarcoidosis with 200 mg daily minocycline over a median 12-month period. With a median followup of 26 months, the authors noted complete and partial responses to treatment in 8 and 2 patients, respectively. The mean time to reach a maximal response was 3.2 months. Of the 8 patients who had a complete response, minocycline could be withdrawn in 7 patients, 3 of whom experienced recurrent lesions and received further treatment with 200 mg daily doxycycline. Complete remission was maintained for a mean of 15.3 months. Regression of pulmonary infiltrates and mediastinal lymphadenopathy was noted in the 2 patients with concurrent pulmonary involvement. 

The results of a nationwide questionnaire survey, performed by a Japanese research group in 2005 (reported in Japanese), indicated that antibiotic therapy was effective in 43% of 87 patients with sarcoidosis treated with many kinds of antibiotics, including minocycline, doxycycline, and clarithromycin. Baba and colleagues [67] used minocycline and clarithromycin for therapy against worsening of multiple endobronchial mass lesions, given the possible roles of *P. acnes* in the pathophysiology of sarcoidosis and the uncertainty of the long-term effects of corticosteroids. The lesions were worsening before the antibiotic therapy was initiated, so the improvements appeared to be due to the therapy. Park and colleagues [68] reported the first case of ocular and ocular adnexal sarcoidosis treated with minocycline. The patient in this paper demonstrated a complete and recurrence-free response of lacrimal gland and choroidal lesions as well as parotid granulomas and pulmonary infiltrates after 3 months of minocycline therapy. Miyazaki and colleagues [69] reported the regression of nodular lesions of muscular sarcoidosis during minocycline treatment with decreased levels of angiotensin-converting enzyme, lysozyme, and soluble interleukin-2 receptor. The effectiveness of minocycline for muscular sarcoidosis in this case was confirmed when a prompt response to the minocycline therapy was repeatedly observed.

Based on the studies described in this section, the antimicrobial properties of tetracyclines are effective for treating sarcoidosis. Many researchers have questioned the antimicrobial role of tetracyclines because tetracyclines also have anti-inflammatory properties which were demonstrated by *in vitro* studies and corroborated by clinical trials. Tetracycline suppresses neutrophil migration and chemotaxis [70], and minocycline inhibits T-lymphocyte activation and proliferation [71]. Both minocycline and doxycycline obviate granuloma formation *in vitro* [72]. Although it remains controversial whether these antibiotics kill microbes or have only an anti-inflammatory effect, the mechanisms of sarcoid granuloma formation caused by *P. acnes* proposed in this chapter suggest that these antibiotics have many roles in the process of granuloma formation, including the events triggering granuloma formation caused by intracellular proliferation of the bacterium.

The Marshall protocol [73] is an eradication therapy for intracellular bacteria established by Dr. Trevor Marshall. This therapeutic protocol is a combination of minocycline plus azithromycin or clindamycin, supported by the use of an angiotensin receptor blocker to prevent Herxheimer reactions. According to the results published by the Autoimmunity Research Foundation in 2006, this therapy is effective in 62% of patients with sarcoidosis. Information about the treatment can be found at the study site, http://marshallprotocol.com/ and also at http://autoimmunityresearch.org/.

## 10. Summary 

*P. acnes* is currently the only microorganism that has been isolated from sarcoid lesions by bacterial culture. In the late 1970s, Homma et al. reported frequent isolation of *P. acnes* from biopsy specimens of Japanese patients with sarcoidosis. In the 1980s, Abe et al. isolated the same microbe from 31 (78%) of 40 sarcoid lymph nodes. These Japanese researchers also cultured this organism from 20% of 141 nonsarcoid lymph nodes. In 1999, Ishige et al. reported many genomes of *P. acnes* in biopsy samples of lymph nodes from 12 of 15 Japanese patients with sarcoidosis. Biopsy samples of the three patients without *P. acnes* all showed many genomes of *P. granulosum*. Similar results were obtained in an international collaborative study of samples from European patients with sarcoidosis. 

In 2002, Yamada et al. found *P. acnes* genomes in and around sarcoid granulomas, suggesting that this indigenous bacterium is related to granulomatous inflammation in sarcoidosis. In 2012, Negi et al. reported that a novel monoclonal antibody specific to *P. acnes* that recognizes lipoteichoic acid of the plasmalemma reacted with small round bodies within sarcoid granulomas in 88% of lymph node biopsy samples, 77% of video-assisted thoracic surgery lung samples, 57% of transbronchial lung biopsy samples from Japanese patients with sarcoidosis, and in 89% of lymph node biopsy samples from German patients with sarcoidosis. Reactivity to the antibody was not observed in nonsarcoid granulomas, including those from patients with tuberculosis and so-called sarcoid reaction. The high frequency and specificity of *P. acnes* detected within sarcoid granulomas indicate that this indigenous bacterium might be the cause of granuloma formation in many patients with sarcoidosis. 

*P. acnes* is the most common commensal bacterium in the lungs and lymph nodes of subjects without sarcoidosis. *P. acnes* is found in 20% of nonsarcoid lymph nodes by bacterial culture and in 15% of nonsarcoid lymph nodes by polymerase chain reaction. In nonsarcoid samples examined by immunohistochemistry, *P. acnes* was found in 18% of lung samples and in 22% of lymph node samples. Occasional detection of *P. acnes* in nongranulomatous areas of the lungs and lymph nodes from nonsarcoid patients indicates that host factors may be more critical than agent factors in the sarcoidosis etiology. 

A particular protein, referred to as a trigger factor, from *P. acnes* causes a cellular immune response in some sarcoid patients, but not in subjects without sarcoidosis. The *P. acnes* trigger factor protein induces pulmonary granulomas in mice sensitized with the protein and adjuvant, but only in mice with latent *P. acnes* infection in their lungs. This pathogenesis was confirmed by further experiments using mice sensitized with *P. acnes* and adjuvant, in which the eradication of *P. acnes* by antibiotics prevented granulomas in this experimental model. The findings from this experimental model of sarcoidosis might explain the recently reported effectiveness of tetracyclines for treating sarcoidosis. 

This indigenous low-virulence bacterium can cause latent infection in the lungs and lymph nodes and persist in a cell-wall-deficient form. This dormant form of *P. acnes* can be activated endogenously under certain environmental conditions and then proliferate in cells at the site of latent infection. In patients who are hypersensitive to this endogenous bacterium, granulomatous inflammation is triggered by intracellular proliferation of the bacterium. Some of the proliferating bacteria may escape from isolation by the granuloma and spread to other organs via the lymphatic and blood streams. The spread of infective *P. acnes* might cause a new latent infection in systemic organs such as the eyes, skin, and heart. Latent infection established in certain systemic organs will be reactivated simultaneously by the next triggering event, resulting in the onset of systemic sarcoidosis.
